# Application of an improved wide–narrow-band hybrid ANC algorithm in a large commercial vehicle cabine

**DOI:** 10.1038/s41598-024-60979-7

**Published:** 2024-05-07

**Authors:** Jintao Su, Jinquan Nie, Shuming Chen

**Affiliations:** 1 School of Automotive and Traffic Engineering, Hubei University of Arts and Science, Xiangyang, 441053 China; 2 Hubei Key Laboratory of Power System Design and Test for Electrical Vehicle, Hubei University of Arts and Science, Xiangyang, 441053 China; 3 Jilin University, Changchun, 130022 China

**Keywords:** Noise control, Notch FxLMS algorithm, Wide-narrowband hybrid ANC, Aerospace engineering, Engineering, Mechanical engineering

## Abstract

In recent years, active noise reduction technology has become a research hotspot. However, most active noise reduction technologies are developed based on passenger vehicles and are not implemented in large commercial vehicles. The large commercial vehicle sound field space is large, the traditional active noise reduction method is difficult to implement. In order to explore this problem, this paper proposes an improved active noise control method for commercial vehicles: (1) based on the traditional notch filter, a notch FxLMS algorithm based on speed smoothing is proposed; (2) on the basis of the traditional wire–narrowband hybrid ANC algorithm, the reference signal weighting technology is introduced into the wide-band subsystem, and the notch FxLMS algorithm based on speed smoothing is used as the narrowband subsystem, so as to propose an improved wire-narrowband hybrid ANC algorithm. With the help of MATLAB, the simulation model of the designed algorithm is established, and the collected commercial vehicle test noise data is used as the reference signal to simulate and verify the proposed algorithm. The results show that the proposed method has certain practicability.

## Introduction

Compared with passive Noise Control technology, Active Noise Control technology (Active Noise Control, ANC) has the advantages of prominent low-frequency Noise Control effect, small volume and lightweight^1–3^. At the same time, the control system can be designed according to the characteristics of noise control, so that the noise control is targeted. Therefore, it is very suitable for controlling low frequency noise. With the rapid development of electronic technology and the deepening of active noise control technology, active noise control technology has entered a period of rapid development. The development of digital signal processor makes it possible to use adaptive control theory to develop active noise controller^4–6^.

In terms of active noise reduction research, some scholars have made positive contributions. Morgan and Burgress^7^ proposed an adaptive active noise control system based on Filtered-x Least Mean Square (FxLMS) algorithm for the first time. The algorithm is studied by computer simulation aiming at pipe noise control. At the same time, the delay of the secondary path of the active noise control system is considered to enhance the system stability. Due to these advantages, FxLMS algorithm has become the most widely used algorithm in ANC system. Oswald^8^ developed a single-channel adaptive automotive active noise control system to control the noise generated by diesel engines. The system uses the engine speed signal to calculate the engine noise frequency, and uses the frequency to construct the reference noise signal related to the engine speed signal. The results show that the system has a good control effect on engine noise below 200 Hz. A scholar from the Institute of Acoustic Vibration of the University of Southampton has developed an adaptive noise active control system for passenger cars, effectively reducing vehicle cab noise^9–11^. Scholars from Nanyang University of Science and Technology in Singapore proposed a new adaptive active noise control method based on equal loudness compensation, which can enhance the perception of real changes in noise level and effectively reduce noise radiation^12,13^. In terms of subjective evaluation of active noise reduction, Spanish scholars analyzed sound quality through psychoacoustic parameter prediction and subjective score, and evaluated the ability of multi-channel active noise control system to obtain more pleasant sound. In the research of active noise control system, adaptive filter and adaptive algorithm are the core. At present, the research of active noise control algorithm is the focus of active noise control technology. Researchers hope to develop an efficient and stable adaptive filtering algorithm^14,15^. Luis Vicente^16^ improved the FxLMS algorithm by setting the upper limit of the maximum stride length. The simulation results show that the convergence speed and stability of the control algorithm are improved. Li^17^ proposed a control strategy optimization method based on Genetic Algorithm (Genetic Algorithm-GA), using Genetic Algorithm as a strategy optimization tool for vehicle ANC simulation model optimization and control strategy development. Mohamded^18^ proposed a new fast transverse filtering algorithm with low complexity and good convergence speed on the basis of double Kalman filtering algorithm, and applied it in the field of stereo echo cancellation. Li^19^ proposed a simplified diagonal structure bilinear filter-x least mean square algorithm and a diagonal structure bilinear filter-x least mean square algorithm. Compared with the traditional least mean square algorithm, this algorithm reduces the computational complexity and improves the computational accuracy. Basant^20^ proposed a Delayed filter-x Least Mean Square-DFxLMS algorithm (delayed filter-x least mean square-DFXLMS). This algorithm takes into account the delay of active noise controller. The experiment verifies that DFxLMS algorithm is superior to FxLMS algorithm (Filter-x Least Mean Square-FxLMS) when the delay time is longer. Radik^21^ extended the tangential hyperbolic function nonlinear filter-x mean square algorithm (Tangential Hyperbolic Function-Nonlinear Filtered-x Least Mean Square—THF-NLFxLMS) to wiener-Hammerstein system and proposed the Wiener-Hammerstein THF-NLFXLMS algorithm. Simulation results show that Wiener–Hammerstein THF-NLFXLMS algorithm has better nonlinear noise reduction effect with the increase of system nonlinearity. Ferrer^22^ applies the incremental cooperation strategy in the network to the Multiple error filtered-x least mean square-MEFxLMS (Multiple Error Filtered-x Least Mean Square-MEFxLMS), and develops an active noise control system for adaptive distributed networks. Simulation results show that the proposed distributed algorithm can achieve good performance when appropriate parameters are selected. Akraminia^23^ proposed an adaptive feedback active noise control system based on wavelet algorithm. At the same time, an adaptive learning algorithm is introduced to maintain the stability and convergence speed of the control algorithm. In a typical linear/nonlinear case, the proposed method is compared with FxLMS algorithm and ANC algorithm based on neural network. The results show that the proposed method has good performance in both convergence rate and noise suppression when the quadratic path filtering is not accurate. Jordan^24^ applied adaptive filtering technology to the multi-input multiple-output active control problem, and proposed the multi-input multiple-output FxLMS algorithm with subband adaptive filtering. The multi-input multi-output FxLMS algorithm with adaptive filtering is compared with the standard all-band algorithm. The results show that compared with the full-band implementation, the computational cost of the adaptive filtering algorithm decreases significantly with the increase of the number of sub-bands. Kim^25^ improved the FuLMS algorithm and applied the ultra-stable adaptive recursive filter into the variable-step LMS algorithm, which improved the stability of the control algorithm without reducing the convergence speed of the algorithm. On the basis of FxLMS algorithm, many scholars normalized the reference signal, thus proposed the normalized least mean square algorithm (Normalized Least Mean Square-NLMS), and improved NLMS algorithm^26–29^, which improved the convergence speed of NLMS algorithm compared with FxLMS algorithm. Xu^30^ studied the algorithm of adaptive filtering recursive least square method (Recursive Least Square-RLS) and input the algorithm into the signal processor. By comparing the experimental results with the simulation results, the effectiveness of using RLS algorithm for active noise control was obtained. Shi^31^ studied the noise cancellation system of RLS algorithm and confirmed the effect of communication speech signal processing through simulation. Ayesha^32^ proposed several active control algorithms based on FxRLS (Filtered-x Recursive Least Square-FxRLS) to actively control impulse noise, one of which is to ensure the stability of the control algorithm by setting a threshold for the reference noise signal and error signal. Simulation results show that the improved control algorithm can solve the problem of poor stability of FxRLS algorithm when dealing with impulse noise. Lu^33^ proposed an active noise control method based on the FxRMC (Filtered-x Recursive Maximum Correntropy—FxRMC), which does not require any prior information of noise and has strong interference to impulse noise. Simulation results and experimental results show that this method has better stability and noise reduction effect. Akraminia applied wavelet algorithm to active noise control^34–36^. The simulation results show that the proposed method has fast convergence speed and good noise reduction effect for nonlinear system active noise control. In order to improve the convergence speed of the active control algorithm and reduce the computation amount of the single iteration process of the control algorithm, the two-dimensional coordinate descent method and affine projection method are also adopted in the active control algorithm. Despite the continuous development of active noise reduction technology in recent years, there are still some problems in noise reduction effect and algorithm, such as the limitations of active noise reduction in frequency, and the limitations of active noise reduction in broadband problems, etc. At the same time, the spatial distance of active noise reduction also affects the noise reduction effect^37–42^.

In order to explore the response mechanism of active noise reduction space distance. In this paper, an improved method of active noise control for commercial vehicles is proposed. On the basis of traditional notch filtering, a notch FxLMS algorithm based on velocity smoothing is proposed. In the narrow band subsystem, a notch FxLMS algorithm based on velocity smoothing is used, and an improved line narrow band hybrid ANC algorithm is proposed. The proposed algorithm is verified by simulation on a real vehicle.

## Classical ANC theory and methods

There are many classification methods for active noise control systems, which can be divided into finite impulse response (FIR) filters and infinite impulse response (IIR) filters according to the different filter structures of the controllers. According to the reference noise signal can be divided into feed-forward structure and feedback structure. According to the number of secondary sound sources and error microphones, it can be divided into single-channel ANC system and multi-channel ANC system. According to the spectral characteristics of the processed noise, it can be divided into wide-band ANC system, narrowband ANC system, and wide-band narrowband hybrid ANC system^3–10^.

### Classical adaptive active noise control algorithm

#### FxLMS algorithm

**Figure 1:**
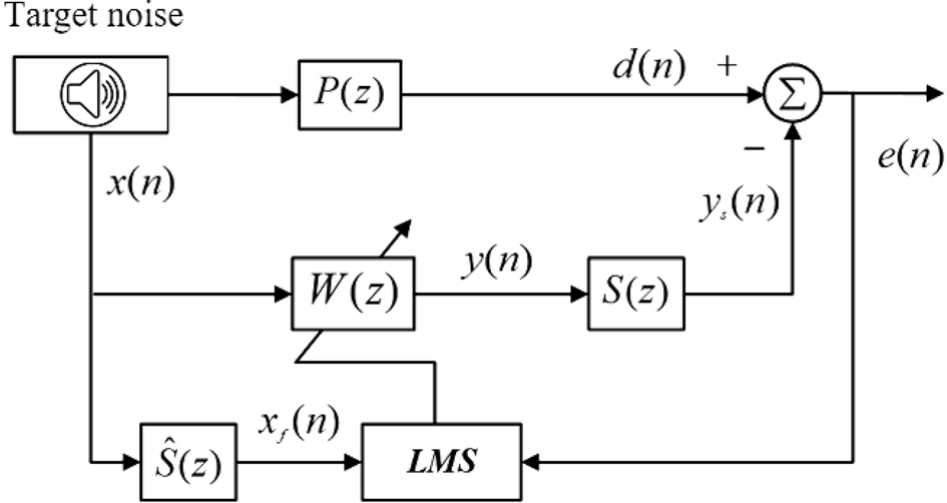
Block diagram of FxLMS control algorithm.

At present, the most widely used adaptive active noise control algorithm is FxLMS algorithm, which is based on LMS algorithm and considers the influence of secondary sound channel. Figure 1 is the block diagram of FxLMS algorithm, *d*(*n*) represents the primary noise signal, *x*(*n*)represents the reference noise signal, *P*(*z*) represents the transfer function of the primary sound channel, *e*(*n*) represents the error signal, *y*(*n*) represents the output signal of the secondary loudspeaker, *S*(*z*) represents the transfer function of the secondary sound channel, $\hat{S}(z)$ represents the estimation of the transfer function of the secondary sound channel. *W*(*z*) represents the transfer function of the adaptive filter^3–6^.

Assuming that the order of ANC controller filter is* L*, then the filter coefficient at time *n* can be expressed as^3–6^:

1 $${\varvec{W}}(n) = \left[ {w_{1} (n),w_{2} (n),w_{3} (n), \ldots ,w_{L} (n)} \right]^{T} .$$

The controller input signal can be expressed as:

2 $${\varvec{X}}(n) = \left[ {x(n),\;x(n - 1),\;x(n - 2), \ldots ,x(n - L + 1)} \right]^{T} .$$

The controller output signal can be expressed as:

3 $$y(n) = {\varvec{X}}^{T} (n){\varvec{W}}(n) = \sum\limits_{i = 1}^{L} {w_{i} } (n)x(n - i + 1).$$

Assuming that the secondary acoustic channel function is estimated by a filter of order *M*, the filter coefficient at time *n* can be expressed as^4–6^:

4 $${\varvec{S}}(n) = \left[ {s_{1} (n),s_{2} (n),s_{3} (n), \ldots ,s_{M} (n)} \right]^{T} .$$

The error noise signal can be expressed as:

5 $$\begin{aligned} e(n) = & d(n) + \sum\limits_{m = 1}^{M} {s_{m} } (n)y(n - m + 1) \\ = & d(n) + \sum\limits_{m = 1}^{M} {s_{m} } (n){\varvec{X}}^{T} (n - m + 1){\varvec{W}}(n - m + 1) \\ = & d(n) + {\varvec{X}}_{f}^{T} (n){\varvec{W}}(n), \\ \end{aligned}$$

where ${\varvec{X}}_{f} (n)$ is the reference noise signal processed by the secondary acoustic channel filtering:

6 $${\varvec{X}}_{f} (n) = \left[ {x_{f} (n),x_{f} (n - 1),x_{f} (n - 2), \ldots ,x_{f} (n - L + 1)} \right]^{T} ,$$

7 $$x_{f} (n) = \sum\limits_{m = 1}^{M} {s_{m} } (n)x(n - m + 1).$$

FxLMS algorithm follows the minimum mean square error criterion, taking the mean square error of the error noise signal value as the objective function, and its expression is:

8 $$J(n) = E\left[ {e^{2} (n)} \right],$$

where, $E\left[ \cdot \right]$ represents time averaging of independent variables:

Substitute Eq. (5) into Eq. (8) to obtain:

9 $$\begin{aligned} J(n){ = } & E\left[ {d^{2} (n)} \right] + {\varvec{W}}^{T} (n)E\left[ {{\varvec{X}}_{f}^{{}} (n){\varvec{X}}_{f}^{T} (n)} \right]{\varvec{W}}(n) + 2{\varvec{W}}^{T} (n)E\left[ {d(n){\varvec{X}}_{f}^{T} (n)} \right] \\ = & E\left[ {d^{2} (n)} \right] + {\varvec{W}}^{T} (n){\varvec{RW}}(n) + 2{\varvec{W}}^{T} (n)\user2{P,} \\ \end{aligned}$$

10 $${\varvec{R}} = E\left[ {{\varvec{X}}_{f}^{{}} (n){\varvec{X}}_{f}^{T} (n)} \right],$$

11 $${\varvec{P}} = E\left[ {d(n){\varvec{X}}_{f}^{T} (n)} \right].$$

According to Eq. (9), *J(n)* is a quadratic function of the weight vector ***W****(n)*, while ***R*** is a positive definite quadratic matrix. Therefore, there is a unique minimum point. When *J(n)* achieves the minimum value, the optimal value ***W***_*o*_ of weight coefficient satisfies:

12 $${\varvec{W}}_{o} = - {\varvec{R}}^{ - 1} {\varvec{P}}\user2{.}$$

It is too expensive to directly solve the filter weight coefficient of ANC controller according to Eq. (12). In order to avoid matrix operation, a recursive estimation algorithm is introduced to calculate the filter weights iteratively. According to the steepest descent method, the recursive relation of filter weight coefficients can be obtained as follows:

13 $${\varvec{W}}(n + 1) = {\varvec{W}}(n) - \frac{\mu }{2}\nabla (n).$$

In Eq. (13), *μ* represents the convergence factor, which is used to regulate the convergence speed and stability of the algorithm. $\nabla (n)$ is the gradient vector and its expression is:

14 $$\nabla (n) = \frac{\partial J(n)}{{\partial {\varvec{W}}(n)}} = \frac{{\partial e^{2} (n)}}{{\partial {\varvec{W}}(n)}} = 2e(n){\varvec{X}}_{f} (n).$$

Therefore, the iterative formula of filter weight coefficient of FxLMS algorithm is expressed as:

15 $${\varvec{W}}(n + 1) = {\varvec{W}}(n) - \mu e(n){\varvec{X}}_{f} (n).$$

In practice, the value of ${\varvec{X}}_{f} (n)$ cannot be obtained directly and is usually represented by its estimator ${\varvec{X}}_{f}^{\prime} (n)$. ${\varvec{X}}_{f}^{\prime} (n)$ is obtained by using the secondary acoustic channel estimation function to process the reference noise signal, and the secondary acoustic channel transfer function needs to be identified and replaced before it is obtained. Therefore:

16 $${\varvec{W}}(n + 1) = {\varvec{W}}(n) - \mu e(n){\varvec{X}}_{f}^{\prime} (n),$$

17 $${\varvec{X}}_{f}^{\prime} (n) = \left[ {x_{f}^{\prime} (n),x_{f}^{\prime} (n - 1),x_{f}^{\prime} (n - 2), \ldots ,x_{f}^{\prime} (n - L + 1)} \right]^{T} ,$$

18 $$x_{f}^{\prime} (n) = {\varvec{X}}^{T} (n){\varvec{S}}^{\prime} (n) = \sum\limits_{m = 1}^{M} {s_{m}^{\prime} } (n)x(n - m + 1).$$

In order to ensure the gradual convergence of the algorithm in the iterative process, it is necessary to restrict the convergence factor. The range of convergence factor is:

19 $$0 < \mu < \frac{1}{{tr\left[ {{\varvec{R}}_{f} } \right]}},$$

where $tr\left[ \cdot \right]$ represents matrix trace operation, and the specific calculation formula can be expressed as:

20 $$tr\left[ {{\varvec{R}}_{f} } \right] = \sum\limits_{j = 1}^{M} {\left( {x_{f}^{\prime} \left( {n - j + 1} \right)} \right)^{2} } .$$

Combined with Eqs. (19) and (20), with the increase of the order of the adaptive filter, the value range of the convergence factor gradually decreases, and the higher order adaptive filter of the algorithm is more likely to diverge.

## Design of improved active noise control algorithm

The noise in the cab of commercial vehicle includes narrowband noise such as engine order noise and wide-band noise such as road noise and wind noise. In order to achieve good active noise control effect, an improved active noise control algorithm is proposed in this paper. One is based on the traditional notch filter, the speed signal smoothing module is introduced, and the speed smoothing based notch wave FxLMS algorithm is proposed to achieve a good control of the second order noise of commercial vehicle engine. The other is an improved hybrid ANC algorithm. The wide-band subsystem uses FxLMS/F algorithm based on reference model weighting, while the narrowband subsystem uses the notch FxLMS algorithm based on speed smoothing to effectively control the narrowband noise such as engine order noise and the wide-band noise such as road noise and wind noise. In this chapter, the theoretical derivation of the two improved algorithms is given.

### Design of notch FxLMS algorithm based on speed smoothing

**Figure 2:**
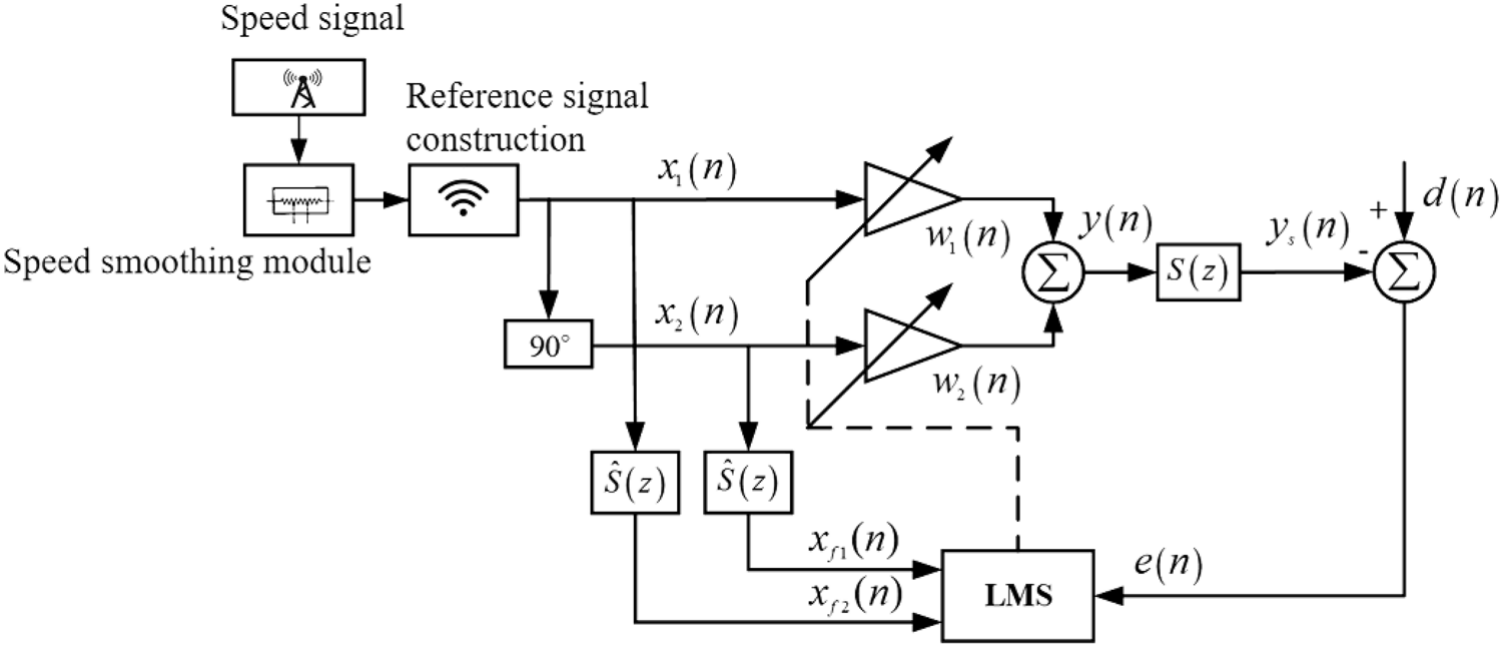
Notch FxLMS algorithm based on speed smoothing.

Based on the theory of adaptive filtering algorithm and the traditional notch algorithm, a notch algorithm FxLMS based on the speed smoothing is proposed by introducing the speed smoothing technique. Figure 2 shows the block diagram of notch FxLMS algorithm based on speed smoothing.

**Figure 3:**
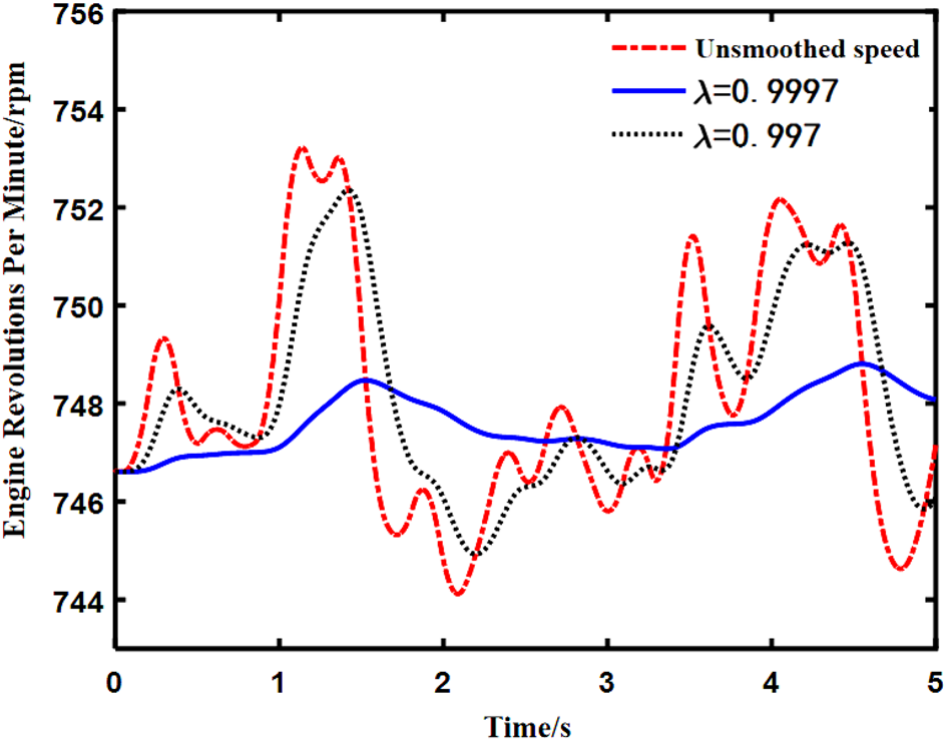
Smoothing effect of engine speed.

In Fig. 2, *x*_1_(*n*) and *x*_2_(*n*) respectively represent two reference signals constructed according to the engine speed. *x*_*f*1_(*n*) and *x*_*f*2_(*n*) respectively represent the two reference signals filtered by the secondary pathway. *w*_1_(*n*) and *w*_2_(*n*) represent the sine and cosine weights of notch filter, respectively. *d*(*n*) represents the noise signal transmitted to the error microphone, which is called the expected noise; *y*(*n*) represents the output signal from the controller to the loudspeaker. *S*(*z*) represents the transfer function of the secondary pathway, $\hat{S}(z)$ represents the estimation of *S*(*z*), *y*_*s*_(*n*) represents the loudspeaker output signal transmitted to the error microphone, and *e*(*n*) represents the signal collected by the error microphone, which is the superposition signal of *d*(*n*) and *y*_*s*_(*n*). The engine order noise of commercial vehicles usually satisfies the following relation:

21 $$f_{i} = \frac{R(n)i}{{60\tau }}\eta ,$$

where *i* is the number of engine cylinders, *R*(*n*) is the engine speed, *τ* is the number of engine strokes, and *η* is the harmonic order. According to the above equation, the engine order frequency corresponding to a specific speed can be calculated, and the reference signal can be constructed accordingly. The traditional ANC algorithm based on notch filter directly uses the collected speed signal to construct the reference signal. However, when the engine speed fluctuates greatly, the variable speed signal will produce the variable reference signal. To solve this problem, Eq. (22) is used to smooth the speed signal:

22 $$R(n) = \lambda R(n - 1) + (1 - \lambda )r(n),$$

where *R*(*n*) is the smooth speed value at time *n*, *R*(*n* − 1) is the smooth speed value at the previous time, and *r*(*n*) is the original speed value of the engine at the current time. $\lambda$ is the forgetting factor, and its value is close to 1. Figure 3 shows the effect of engine speed smoothing in idle driving condition with different values of $\lambda$.

It can be seen from Fig. 3 that the forgetting factor $\lambda$ controls the smoothness of engine speed. If $\lambda$ is close to 1, the better the smoothness is. But a larger $\lambda$ also means it is harder to track changes in the true engine speed.

After calculating the smoothed speed *R*(*n*), the reference frequency signal at the *n*th time is:

23 $$f_{i} = \frac{r(n)i}{{60\tau }}\eta .$$

The controller reference signal constructed at time* n* is:

24 $$\left\{ \begin{gathered} x_{1} (n) = A\sin(2\pi f_{R} (n)) \hfill \\ x_{2} (n) = A\cos (2\pi f_{R} (n)) \hfill \\ \end{gathered} \right..$$

The output signal of the controller at time *n* can be expressed as:

25 $$y(n) = w_{1} (n)x_{1} (n) + w_{2} (n)x_{2} (n),$$

where the notch filter weight coefficients *w*_1_(*n*) and *w*_2_(*n*) are updated based on LMS algorithm. In notch FxLMS algorithm, sine component weight filter and cosine component weight filter usually adopt transverse FIR filter with order 1. According to the least mean square error criterion, the updated formula of sine component weight coefficient and cosine component weight coefficient can be obtained:

26 $$\left\{ {\begin{array}{*{20}c} {w_{1} (n + 1) = w_{1} (n) + 2\mu e(n)x_{f1} (n)} \\ {w_{2} (n + 1) = w_{2} (n) + 2\mu e(n)x_{f2} (n)} \\ \end{array} } \right.,$$

where *μ* is the step size of the algorithm. Filtered reference signals *x*_*f*1_(*n*) and *x*_*f*2_(*n*) are calculated as follows:

27 $$\left\{ {\begin{array}{*{20}c} {x_{f1} (n) = \hat{\user2{S}}^{T} (n){\varvec{x}}_{1} (n)} \\ {x_{f2} (n) = \hat{\user2{S}}^{T} (n){\varvec{x}}_{2} (n)} \\ \end{array} } \right.,$$

where $x_{1} (n) = [x_{1} (n) \, x_{1} (n - 1) \, \cdots \, x_{1} (n - M + 1)]^{T}$ is the reference signal sequence of sine component, $x_{2} (n) = [x_{2} (n) \, x_{2} (n - 1) \, \cdots \, x_{2} (n - M + 1)]^{T}$ is the reference signal sequence of cosine component. *M* is the filter length used to estimate the filter of the secondary pathway. Table 1 shows the calculation flow of notch FxLMS algorithm based on speed smoothing.

**Table 1:** Calculation flow of notch FxLMS algorithm based on speed smoothing.

Initialization	*w*_1_(0) = *w*_2_(0) = 0, ***x***_1_(0) = ***x***_*2*_(0) = **0**, *x*_*f*1_(*n*) = *x*_*f2*_(*n*) = 0, *R*(0) = *r*(0)
Calculation process	For n = 0, 1, 2, … Revolving speed signal *r*(*n*), and error microphone signal *e*(*n*) at the *n*th moment are collected $R(n) = \lambda R(n - 1) + (1 - \lambda )r(n)$ $f_{i} = \frac{r(n)i}{{60\tau }}\eta$ $$\left\{ \begin{gathered} x_{1} (n) = Asin(2\pi f_{R} (n)) \hfill \\ x_{2} (n) = A\cos (2\pi f_{R} (n)) \hfill \\ \end{gathered} \right.$$ $y(n) = w_{1} (n)x_{1} (n) + w_{2} (n)x_{2} (n)$ $$\left\{ {\begin{array}{*{20}c} {x_{f1} (n) = \hat{\user2{S}}^{T} (n){\varvec{x}}_{1} (n)} \\ {x_{f2} (n) = \hat{\user2{S}}^{T} (n){\varvec{x}}_{2} (n)} \\ \end{array} } \right. \,$$ $$\left\{ {\begin{array}{*{20}c} {w_{1} (n + 1) = w_{1} (n) + 2\mu e(n)x_{f1} (n)} \\ {w_{2} (n + 1) = w_{2} (n) + 2\mu e(n)x_{f2} (n)} \\ \end{array} } \right. \,$$ Output control signal *y*(*n*) End

### Design of improved active noise control algorithm for broadband and narrowband hybrid

In commercial vehicle driving, there will be not only engine order noise, but also road noise, wind noise and other broadband noise. Therefore, the broadband noise must be controlled. In this section, an improved wide-narrow-band mixed ANC algorithm is proposed. The algorithm consists of broadband subsystem, narrowband subsystem and signal separation subsystem. Compared with the traditional width-narrowband hybrid algorithm, the improved width-narrowband hybrid algorithm introduces speed smoothing module in the narrowband subsystem, and FxLMS/F algorithm based on reference signal weighting is introduced in the broadband subsystem. The block diagram of the improved broadband and narrowband hybrid ANC algorithm is shown in Fig. 6. The detailed introduction of each subsystem in the improved hybrid algorithm is as follows.

#### Signal separation (SNC) subsystem

In the broadband and narrowband ANC algorithm, the function of the signal separation subsystem is to remove the narrowband component in the noise signal collected by the reference microphone, so as to realize the effective separation of the broadband and narrowband components in the broadband narrowband mixed noise, and improve the denoising effect of the broadband subsystem. In the improved broadband hybrid ANC algorithm, the SNC subsystem is used to separate the second-order noise from the cab noise, and the remaining noise signals are processed by the broadband subsystem.

**Figure 4:**
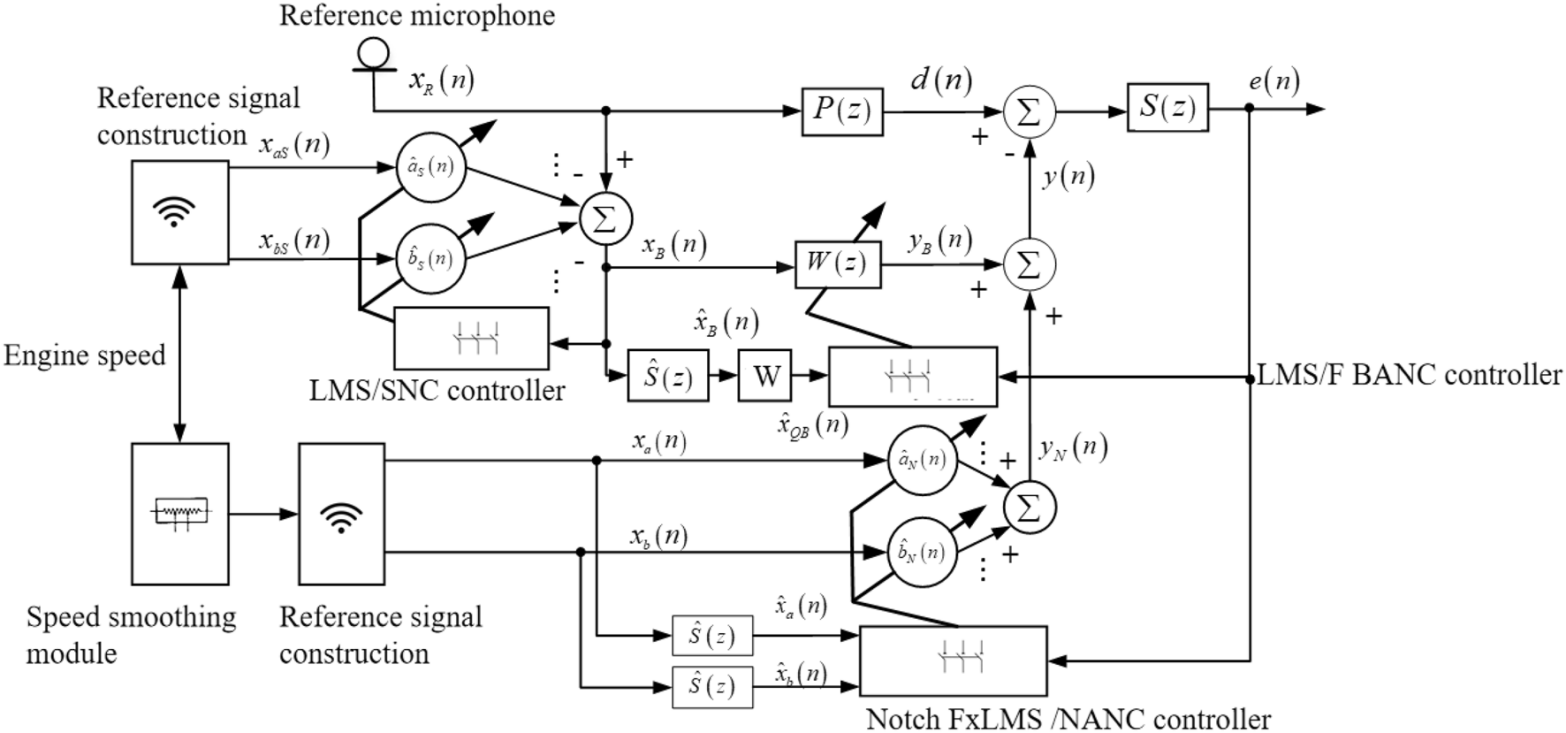
Improved wide-narrowband mixed ANC algorithm.

As shown in Fig. 4, the input signal of the SNC subsystem is solved by the engine speed. Assuming the engine speed is *r*(*n*), the corresponding reference frequency signal is expressed as:

28 $$f(n) = \frac{r(n)i}{{60\tau }}\eta .$$

The constructed reference signal of SNC subsystem is expressed as:

29 $$\left\{ {\begin{array}{*{20}c} {x_{aS} = \sin (2\pi f(n)n)} \\ {x_{bS} = \cos (2\pi f(n)n)} \\ \end{array} } \right..$$

The SNC subsystem uses LMS algorithm to update the filter weights. The output signal of SNC subsystem can be expressed as^7–12^:

30 $$y_{SNC} (n) = \hat{a}_{S} (n)x_{aS} (n) + \hat{b}_{S} (n)x_{bS} (n),$$

where $\hat{a}_{S} (n)$ and $\hat{b}_{S} (n)$ represent the filter weight coefficients of sine and cosine components respectively, and their updates follow the following formula:

31 $$\left\{ {\begin{array}{*{20}c} {\hat{a}_{S} (n + 1) = \hat{a}_{S} (n) + \mu_{S} e_{S} (n)x_{aS} (n)} \\ {\hat{b}_{S} (n + 1) = \hat{b}_{S} (n) + \mu_{S} e_{S} (n)x_{bS} (n)} \\ \end{array} } \right.,$$

where $\mu_{s}$ is the step size of the SNC subsystem, $e_{s} (n)$ is the separation error of the SNC subsystem, and the input reference signal $x_{B} (n)$ of the broadband subsystem is expressed as:

32 $$x_{B} (n) = e_{S} (n) = x_{R} (n) - \hat{a}_{S} (n)x_{aS} (n) - \hat{b}_{S} (n)x_{bS} (n).$$

With the continuous iteration of the filter weight coefficient, the engine order noise component in the vehicle noise is gradually separated, and the separation error $e_{s} (n)$ gradually becomes the broadband component of the vehicle noise.

#### Narrowband (NANC) subsystem based on speed smooth notch FxLMS algorithm

In the wide-narrow-band hybrid ANC algorithm, the narrow-band subsystem controls the engine order noise in the cab, and its reference signal is obtained from the engine speed signal after smoothing^10–14^:

33 $$\left\{ \begin{gathered} x_{a} (n) = Asin(2\pi f_{R} (n)) \hfill \\ x_{b} (n) = A\cos (2\pi f_{R} (n)) \hfill \\ \end{gathered} \right..$$

The output of the NANC subsystem is expressed as:

34 $$y_{N} (n) = \hat{a}_{N} (n)x_{a} (n) + \hat{b}_{N} (n)x_{b} (n).$$

The weight update formula of NANC subsystem is expressed as:

35 $$\left\{ {\begin{array}{*{20}c} {\hat{a}_{N} (n + 1) = \hat{a}_{N} (n) + 2\mu_{N} e(n)\hat{x}_{a} (n)} \\ {\hat{b}_{N} (n + 1) = \hat{b}_{N} (n) + 2\mu_{N} e(n)\hat{x}_{b} (n)} \\ \end{array} } \right..$$

The two filtered reference signals in the formula are calculated by the following formula:

36 $$\left\{ {\begin{array}{*{20}c} {\hat{x}_{a} (n) = \hat{\user2{S}}^{T} (n){\varvec{x}}_{a} (n)} \\ {\hat{x}_{b} (n) = \hat{\user2{S}}^{T} (n){\varvec{x}}_{b} (n)} \\ \end{array} } \right.{,}$$

式中, $${\varvec{x}}_{a} (n) = [x_{a} (n) \, x_{a} (n - 1) \, \cdots \, x_{a} (n - M + 1)]^{T}$$, $${\varvec{x}}_{b} (n) = [x_{b} (n) \, x_{b} (n - 1) \, \cdots \, x_{b} (n - M + 1)]^{T}$$.

#### Broadband ANC subsystem based on FxLMS/F algorithm with reference signal weighting

The broadband subsystem of traditional wide-narrowband hybrid ANC algorithm adopts FxLMS algorithm. However, when the algorithm step size is large, the steady state noise reduction will be reduced, and when the step size is small, the convergence speed will be slow. To solve this problem, variable step size strategy is a better solution. The Filtered-x Least Mean Square/Fourth (Filtered-x Least Mean Square/Fourth-FxLMS/F) algorithm adopts threshold parameters to adjust the step size, so that the algorithm takes a larger step size in convergence and a smaller step size in steady-state. Thus, faster convergence speed and better steady-state noise reduction can be achieved. Firstly, the FxLMS/F algorithm is derived.

The cost function of FxLMS/F algorithm is defined as follows:

37 $$J(n) = \frac{1}{2}e^{2} (n) - \frac{1}{2}\phi \ln (e^{2} (n) + \phi ),$$

where *ϕ* is the threshold parameter, and *e*(*n*) is the error signal. The value of *ϕ* determines the convergence ability and steady-state noise reduction ability of the algorithm. According to the above cost function, the filter weight updating formula of FxLMS/F algorithm can be derived by using the steepest gradient descent method:

38 $${\varvec{w}}(n + 1) = {\varvec{w}}(n) + \mu \frac{{e^{2} (n)}}{{e^{2} (n) + \phi }}e(n)\user2{x^{\prime}}(n),$$

where ${\varvec{w}}(n)$ is the filter weight coefficient vector, and $\user2{x^{\prime}}(n)$ is the filter reference signal.

For FxLMS and FxLMS/F algorithm, in the process of filter weight coefficient iteration, the reference signal sampling at each time has the same effect. However, in the process of commercial vehicle driving, the road noise and wind noise have strong random characteristics. Therefore, at any time in the process of ANC control, the reference signal sampling closer to the time can better reflect the real-time variation trend of noise, so more weight is given. Based on this consideration, an FxLMS/F algorithm based on reference signal weighting is used in the broadband subsystem in this study to effectively control the broadband noise in the cab of commercial vehicles. The output signal of the broadband subsystem is calculated by the following formula:

39 $$y_{B} (n) = {\varvec{w}}(n){\varvec{x}}_{B} (n),$$

where ${\varvec{w}}(n) = \left\{ {w_{j} (n)} \right\}_{j = 0}^{L - 1}$ is the weight coefficient vector of the broadband subsystem filter,${\varvec{x}}_{B} (n) = [x_{B} (n) \, x_{B} (n - 1) \, \cdots \, x_{B} (n - L + 1)]^{T}$ is the reference signal vector of the broadband subsystem. The weights of the broadband subsystem filters are updated by the FxLMS/F algorithm weighted by the reference signal, which is expressed as follows:

40 $${\varvec{w}}(n + 1) = {\varvec{w}}(n) + \mu \frac{{e^{2} (n)}}{{e^{2} (n) + \phi }}e(n)\hat{\user2{x}}_{QB} (n),$$

41 $$\hat{\user2{x}}_{QB} (n) = {\varvec{V}} \circ \hat{\user2{x}}_{B} (n),$$

where “$\circ$” is the Hadamard product, $\hat{\user2{x}}_{B} (n) = [\hat{x}_{B} (n) \, \hat{x}_{B} (n - 1) \, \cdots \, \hat{x}_{B} (n - L + 1)]^{T}$ is the filtering reference signal vector, where, $\hat{x}_{B} (n) = \hat{\user2{S}}^{T} (n){\varvec{x}}_{B} (n)$, ***V*** is the weighted vector of the filtered reference signal, which can be expressed as the following vector:

42 $${\varvec{V}} = [\underbrace {1,1, \ldots }_{{L_{0} }}\underbrace {b,b, \ldots }_{{L - L_{0} }}],$$

where *b* is a constant less than 1, and *L*_0_ is an integer between 0 and the order *L* of the broadband filter. To solve the output of the narrowband subsystem and the broadband subsystem, the output of the whole wide-narrowband hybrid ANC algorithm can be expressed as:

43 $$y(n) = y_{N} (n){ + }y_{B} (n).$$

The calculation flow of the proposed improved wire-narrowband hybrid ANC algorithm is shown in Table 2.

**Table 2:** Calculation process of improved broadband and narrowband hybrid ANC algorithm.

Initialization	$\hat{a}_{S} \left( 0 \right) = \hat{b}_{S} \left( 0 \right) = \hat{a}_{N} \left( 0 \right) = \hat{b}_{N} \left( 0 \right) = 0,{\varvec{w}}(0) = {\varvec{x}}_{a} (0) = {\varvec{x}}_{b} (0) = {\varvec{x}}_{B} (0) = {\mathbf{0}},R(0) = r(0)$
Calculation process	For *n* = 0, 1, 2, … Revolving speed signal *r*(*n*), reference microphone signal *x*_*R*_(*n*) and error microphone signal *e*(*n*) at the nth moment are collected $R(n) = \lambda R(n - 1) + (1 - \lambda )r(n)$ $f(n) = \frac{r(n)i}{{60\tau }}\eta$ $f_{R} (n) = \frac{R(n)i}{{60\tau }}\eta$ $$\left\{ {\begin{array}{*{20}c} {x_{aS} = \sin (2\pi f(n)n)} \\ {x_{bS} = \cos (2\pi f(n)n)} \\ \end{array} } \right.$$ $y_{SNC} (n) = \hat{a}_{S} (n)x_{aS} (n) + \hat{b}_{S} (n)x_{bS} (n)$ $x_{B} (n) = e_{S} (n) = x_{R} (n) - \hat{a}_{S} (n)x_{aS} (n) - \hat{b}_{S} (n)x_{bS} (n)$ $$\left\{ {\begin{array}{*{20}c} {\hat{a}_{S} (n + 1) = \hat{a}_{S} (n) + \mu_{S} e_{S} (n)x_{aS} (n)} \\ {\hat{b}_{S} (n + 1) = \hat{b}_{S} (n) + \mu_{S} e_{S} (n)x_{bS} (n)} \\ \end{array} } \right.$$ $$\left\{ \begin{gathered} x_{a} (n) = Asin(2\pi f_{R} (n)) \hfill \\ x_{b} (n) = A\cos (2\pi f_{R} (n)) \hfill \\ \end{gathered} \right.$$ $y_{N} (n) = \hat{a}_{N} (n)x_{a} (n) + \hat{b}_{N} (n)x_{b} (n)$ $$\left\{ {\begin{array}{*{20}c} {\hat{x}_{a} (n) = \hat{\user2{S}}^{T} (n){\varvec{x}}_{a} (n)} \\ {\hat{x}_{b} (n) = \hat{\user2{S}}^{T} (n){\varvec{x}}_{b} (n)} \\ \end{array} } \right. \,$$ $$\left\{ {\begin{array}{*{20}c} {\hat{a}_{N} (n + 1) = \hat{a}_{N} (n) + 2\mu_{N} e(n)\hat{x}_{a} (n)} \\ {\hat{b}_{N} (n + 1) = \hat{b}_{N} (n) + 2\mu_{N} e(n)\hat{x}_{b} (n)} \\ \end{array} } \right.$$ $y_{B} (n) = {\varvec{w}}(n){\varvec{x}}_{B} (n)$ $\hat{x}_{B} (n) = \hat{\user2{S}}^{T} (n){\varvec{x}}_{B} (n)$ $\hat{\user2{x}}_{QB} (n) = {\varvec{V}} \circ \hat{\user2{x}}_{B} (n)$ ${\varvec{w}}(n + 1) = {\varvec{w}}(n) + \mu \frac{{e^{2} (n)}}{{e^{2} (n) + \phi }}e(n)\hat{\user2{x}}_{QB} (n)$ $y(n) = y_{N} (n){ + }y_{B} (n)$ Output control signal *y*(*n*) End

## Verification of improved active noise control algorithm

In this chapter, the simulation model of the algorithm is established based on MATLAB software, and the collected noise data of the commercial vehicle cab is used as the noise signal to be controlled. The notch FxLMS algorithm based on speed smoothing and the improved wide-narrowband hybrid ANC algorithm are simulated. In order to verify the validity of FxLMS algorithm based on speed smoothing and improved hybrid ANC algorithm. Firstly, the simulation model of FxLMS algorithm based on speed smoothing and the improved wide-narrowband hybrid algorithm is established by MATLAB software. According to the calculation process of notch FxLMS algorithm based on speed smoothing and improved wide-narrow-band hybrid ANC algorithm, the corresponding algorithm script file is written by using m language of MATLAB software. Load test noise data, primary acoustic path coefficient and secondary acoustic path coefficient in MATLAB software workspace.The improved algorithm program is written on the original ANC module to verify the improved algorithm ANC.The purpose of algorithm validation is to develop a new active noise reduction controller in order to improve the defects of existing algorithms.

### Idle speed condition verification

**Figure 5:**
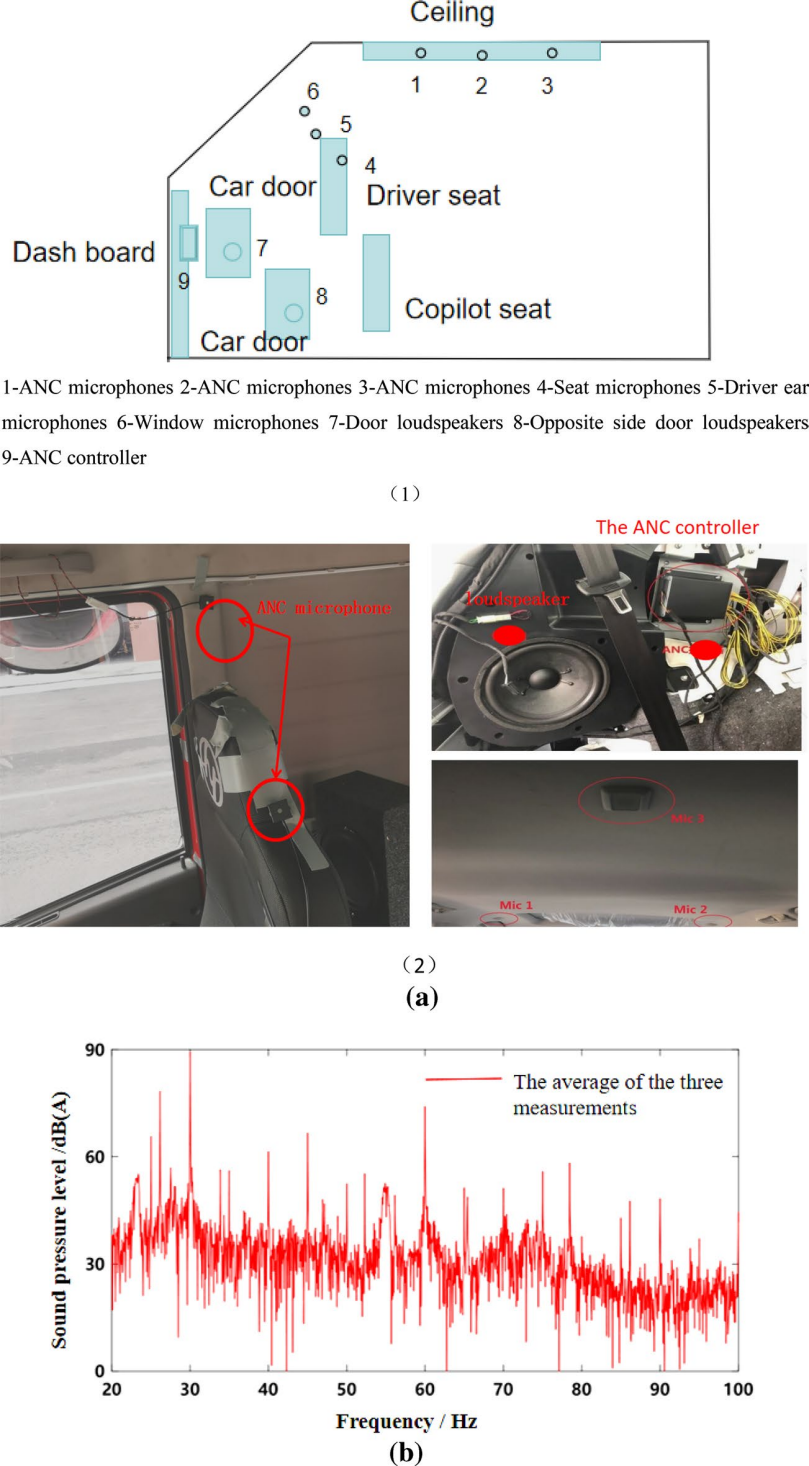
Sensor placement and test results. 1—ANC microphones, 2—ANC microphones, 3—ANC microphones, 4—Seat microphones, 5—Driver ear microphones, 6—Window microphones, 7—Door loudspeakers, 8—Opposite side door loudspeakers, 9—ANC controller. (**a**) ANC test sensor layout. (**b**) Frequency domain curve of interior noise at idle speed.

**Figure 6:**
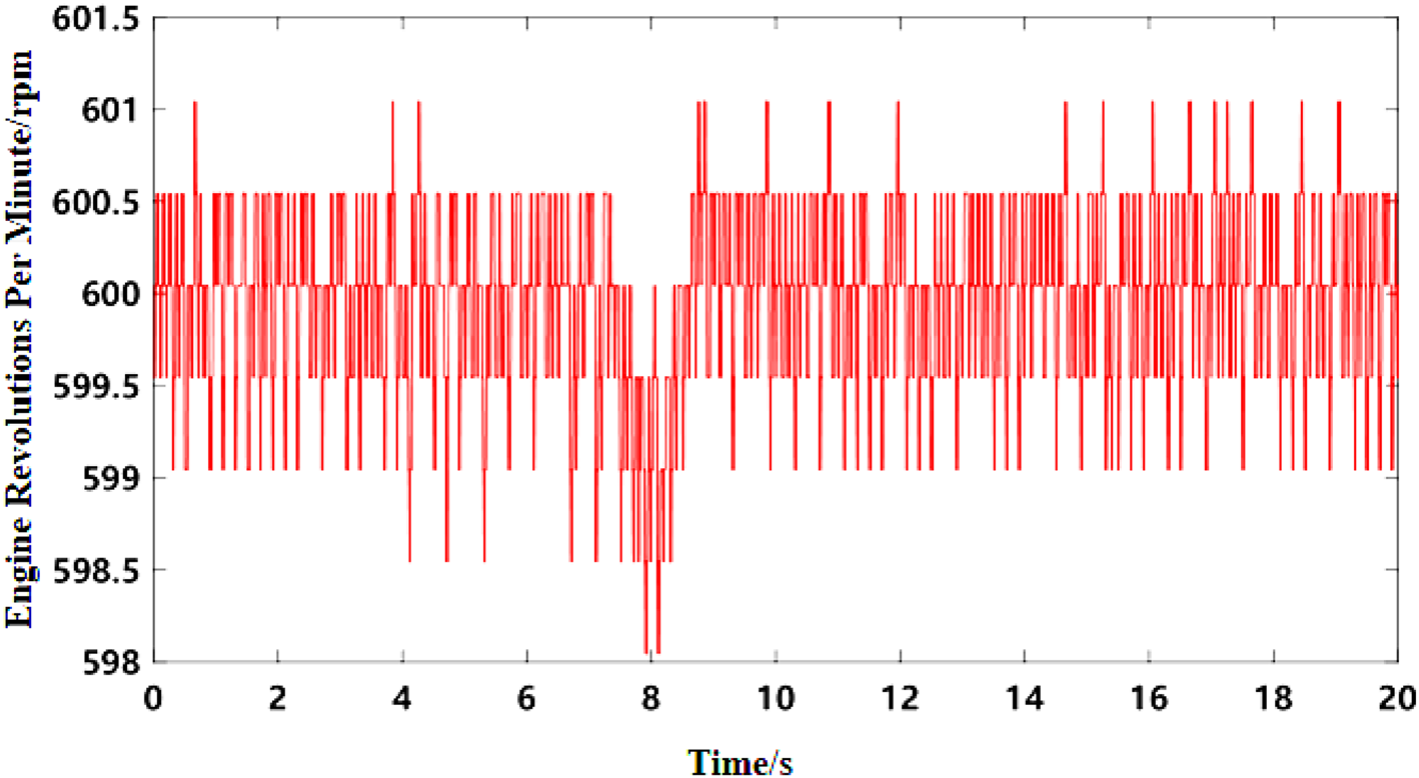
Engine speed curve at idle speed.

A commercial vehicle was used as a test vehicle, which was powered by a six-cylinder four-stroke diesel engine. A standard microphone was arranged in the headrest on the right side of the driver to collect the noise data of the test vehicle in the cab at idle speed. The sensor arrangement is shown in Fig. 5a. ANC control, loudspeaker, ANC microphone and reference point microphone need to be arranged in the cab. Meanwhile, the engine speed is monitored and recorded in real time. The test of engine speed adopts real-time monitoring. The data line is connected to the CAN line of engine speed detection and connected to the acquisition system. During the test, the sampling frequency of data was 51,200 Hz. In terms of experiment, the main path includes reference microphone (Freq range 3.15 Hz to 20 kHz/Sensitivity: 50 mV/Pa) and error microphone (Freq range: 3.15 Hz to 20 kHz/Sensitivity: 50 mV/Pa). The secondary channels include speaker horn, error microphone, speed smoothing module, ANC integrated control module, ANC microphone (Freq range: 3.15 Hz to 20 kHz/Sensitivity: 50 mV/Pa), BANC control module, SNC control module. The above modules are integrated in the overall ANC control module host. ANC controller model ANC-S-E-001 adopts operating voltage 12 V/DC, operating current 200 mA, power 3W, independent integrated module. The loudspeaker adopts the on-board original factory, and the cut-off frequency is 50 Hz.The proposed active noise control algorithm takes engine speed signal as reference. Therefore, the engine speed signal of the test vehicle was collected in the same period, and its sampling frequency was 200 Hz. The spectrum of acquired noise is shown in Fig. 5b, and the engine speed signal collected is shown in Fig. 6.

**Figure 7:**
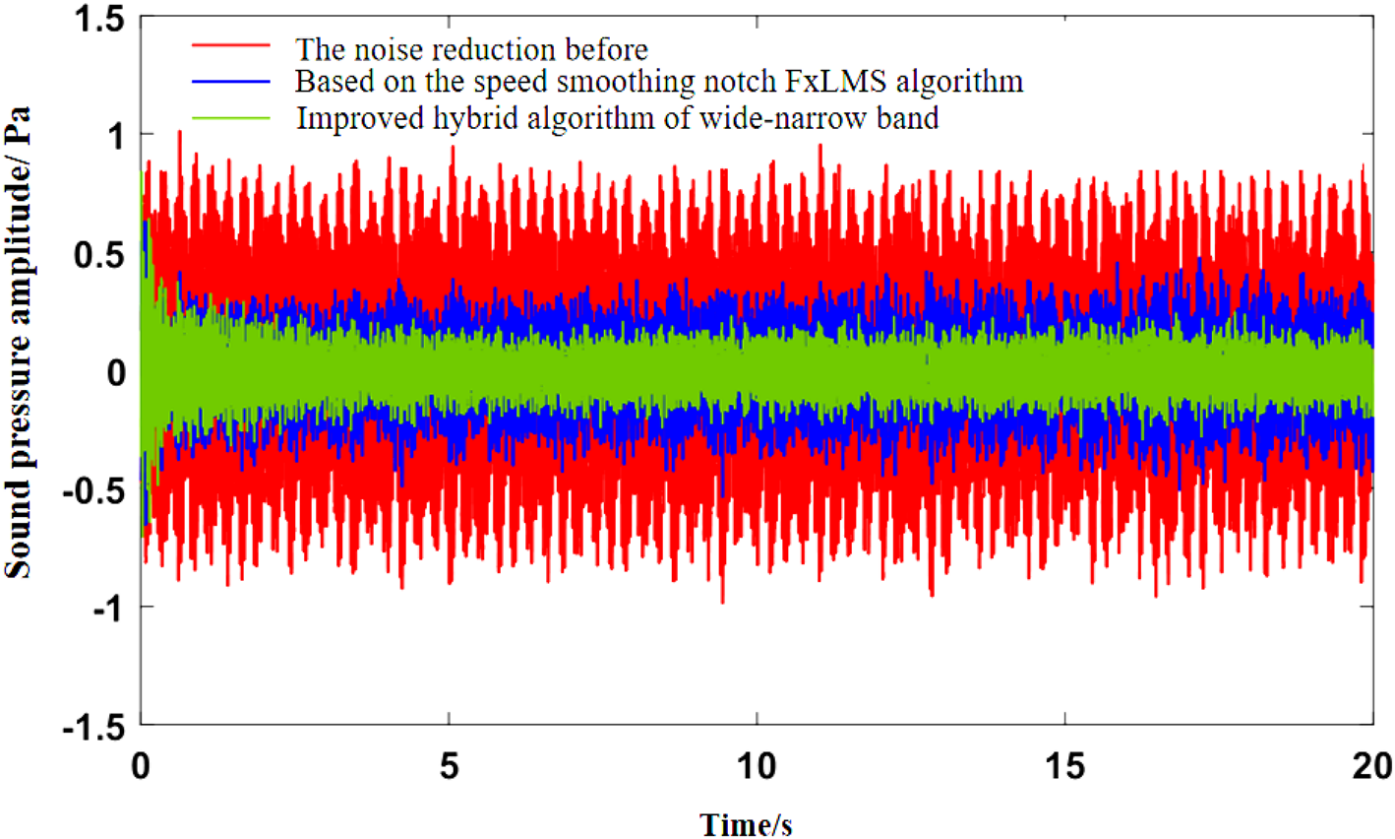
Time-domain noise reduction effect diagram at idle speed.

**Figure 8:**
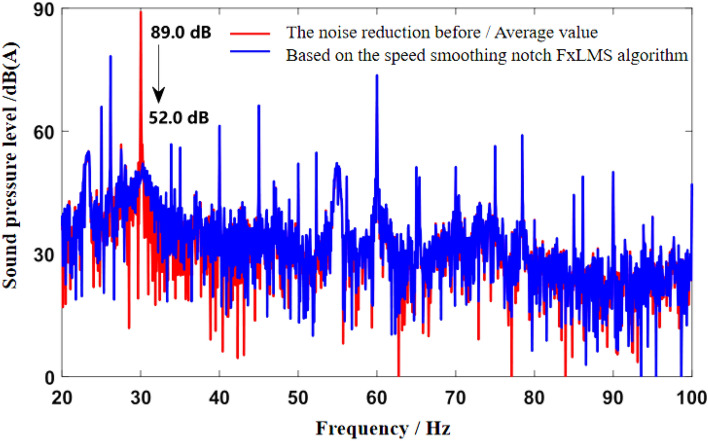
Effect of noise reduction in frequency domain at idle speed (Notch FxLMS algorithm based on speed smoothing).

**Figure 9:**
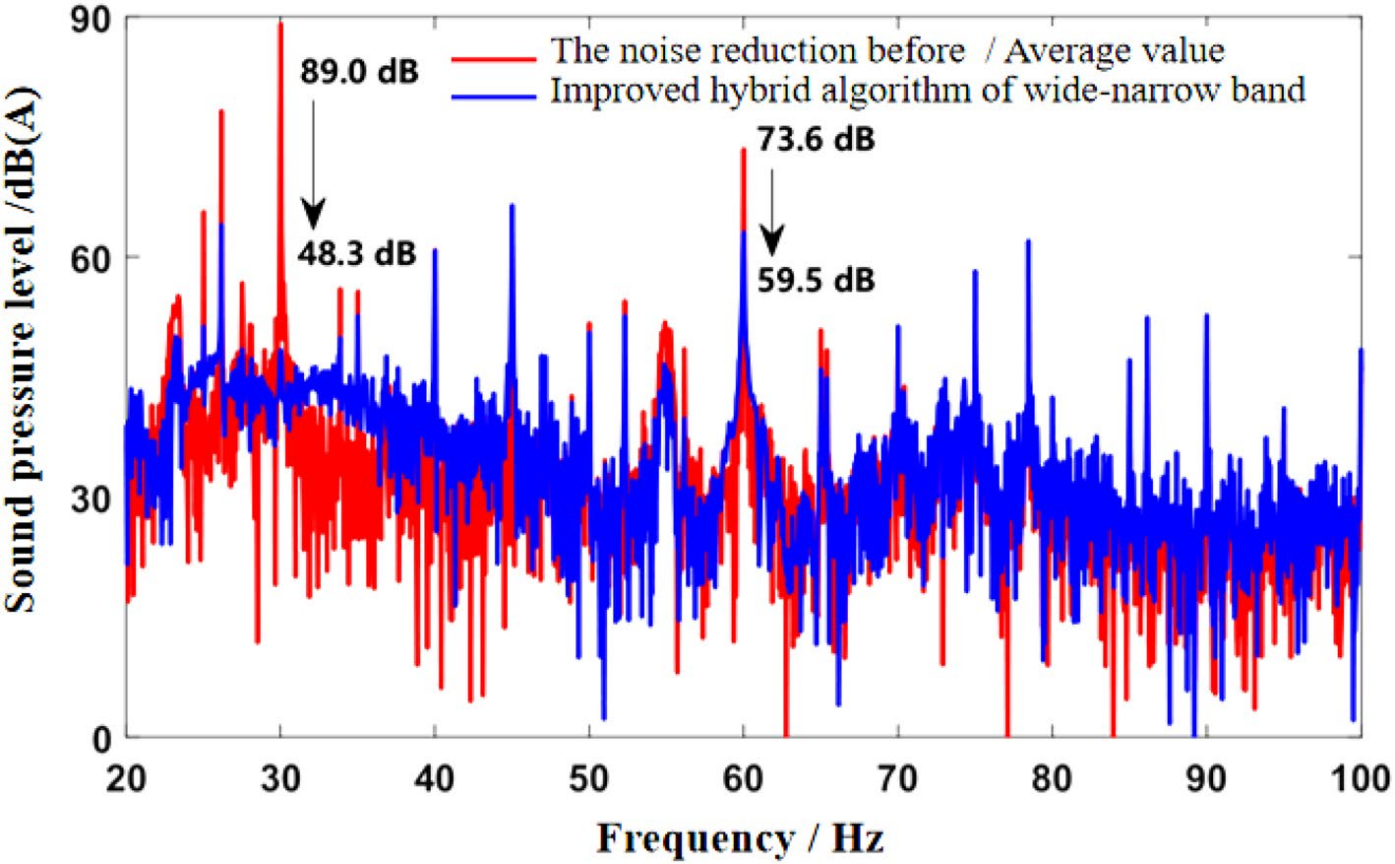
Effect diagram of noise reduction in frequency domain under idling conditions (improved wide-narrow-band hybrid ANC algorithm).

As can be seen from Fig. 5b, the noise frequency at idle speed is mainly the engine’s order noise and the harmonic frequency excited by it. It can be seen from Fig. 6 that the engine speed of the test vehicle at idle speed is 600 r/min, and the calculated frequency of second-order engine noise is 30 Hz, which is consistent with the result in Fig. 5b. In the MATLAB simulation model based on the notch FxLMS algorithm of speed smoothing and the improved wide-narrow-band hybrid ANC algorithm, the active noise control simulation of the collected noise signal is carried out. Considering that the ANC system is limited by the computing capacity of the digital signal processor in practical application, its sampling frequency should not be too high. The sampling frequency used in the simulation is set as 8000 Hz. The selected algorithm parameters are shown in Table 3. The time-domain noise reduction effect is shown in Fig. 7, and the frequency domain noise reduction effect is shown in Figs. 8 and 9.

**Table 3:** Algorithm parameter values in idle speed condition.

Algorithm	Parameters
Notch FxLMS algorithm based on speed smoothing	$\mu = 0.001,\lambda = 0.9999$
Improved wide and narrow band hybrid algorithm	$\mu_{S} = 0.0001,\mu_{N} = 0.001,\mu_{B} = 0.001,\phi = 0.001,b = 0.03,L_{0} = 3,L = 192$

It can be seen from Fig. 7 that both algorithms can quickly converge to the steady state, and the amplitude of sound pressure in the steady state is significantly reduced compared with that before noise reduction. Relatively speaking, the notch FxLMS algorithm based on speed smoothing converges faster than the improved hybrid ANC algorithm. This is because in the broadband and narrowband hybrid algorithm, ANC subsystem needs a convergence process for the separation of broadband signal and narrowband signal, thus reducing the convergence speed of the whole broadband and narrowband hybrid algorithm. From the steady-state point of view, the improved hybrid ANC algorithm has greater noise reduction than the FxLMS algorithm based on speed smoothing. Figure 8 shows that notch FxLMS algorithm based on speed smoothing achieves large noise reduction at the second-order frequency of the engine (30 Hz), and the sound pressure level at 30 Hz drops from 89 dB before noise reduction to 52 dB. As can be seen from Fig. 9, the sound pressure level of the improved broadband-narrowband hybrid ANC algorithm at 30 Hz drops from 89 dB before noise reduction to 48.3 dB, a decrease of 40.7 dB. The improved wide-and-narrow-band hybrid ANC algorithm not only has a good control effect on the second-order noise of the engine at 30 Hz, but also achieves a good noise reduction effect at the fourth-order frequency of the engine (60 Hz) and a band around it. Among them, the sound pressure level at 60 Hz dropped from 73.6 to 59.5 dB, a decrease of 14.1 dB.

As can be seen from the velocity smoothing algorithm in Fig. 8, in the active noise reduction ANC algorithm, the secondary path algorithm has a good effect on the noise reduction processing of narrow band noise. But there are some mismatches in the broadband range. There are some mismatches in the noise reduction effect in the wide frequency range of 40–100 Hz. It is mainly caused by noise signal delay and control signal delay. At the same time, the information sent by the signal generator is not accurate enough, and there will be some mismatch problems. The improved wide and narrow band active control algorithm works well in the low frequency narrow band, but there are some mismatches in the 70–100 Hz wide band, mainly due to the signal error and accuracy of the signal generator. There is also a certain signal delay.

### Uniform driving condition verification

**Figure 10:**
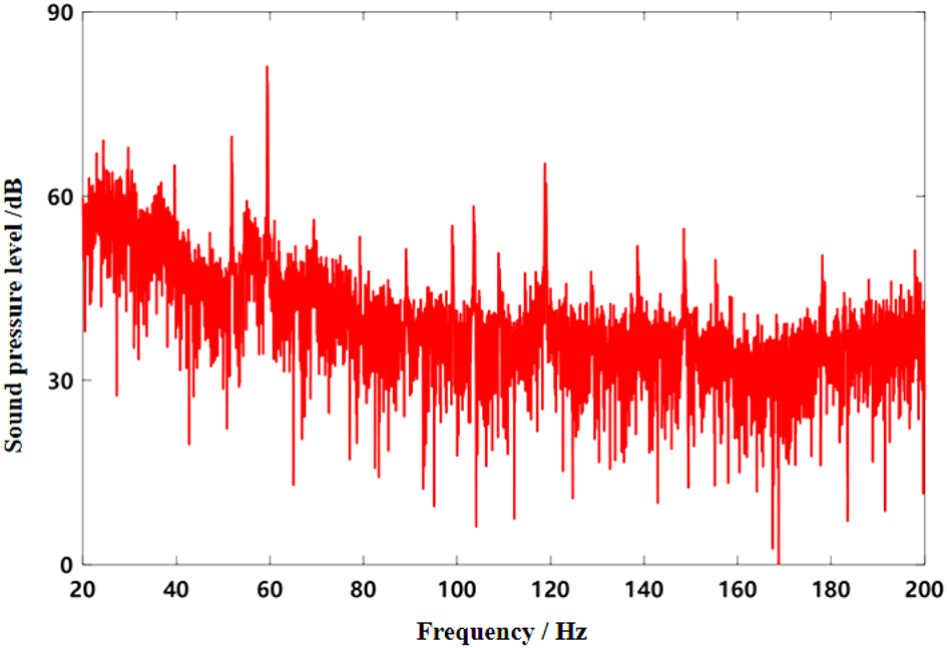
Frequency domain curve of interior noise at 50 km/h constant speed.

**Figure 11:**
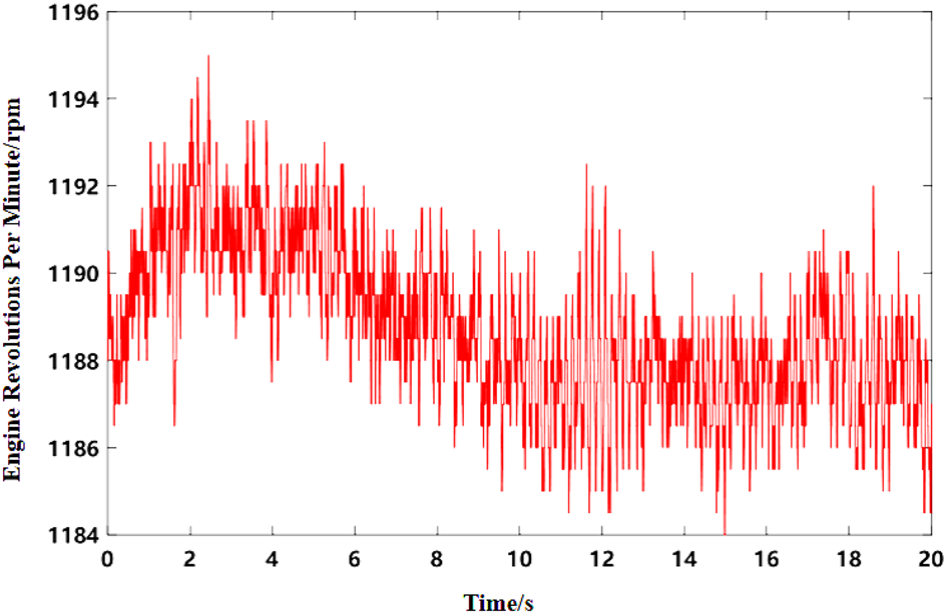
Engine speed curve at 50 km/h constant speed.

The noise and speed data of the test vehicle at 50 km/h constant speed were collected. The spectrum of collected noise is shown in Fig. 10, and the engine speed signal is shown in Fig. 11. As can be seen from Fig. 10, noise at 50 km/h uniform speed includes engine order noise, road noise, wind noise and other components. As can be seen from Fig. 11, the engine speed of the test vehicle fluctuates around 1190 r/min at a constant speed of 50 km/h, and the fluctuation range is about 1184 r/min to 1195 r/min. The calculated frequency of second-order noise of the engine is about 59.5 Hz, which is consistent with the result in Fig. 10.

**Figure 12:**
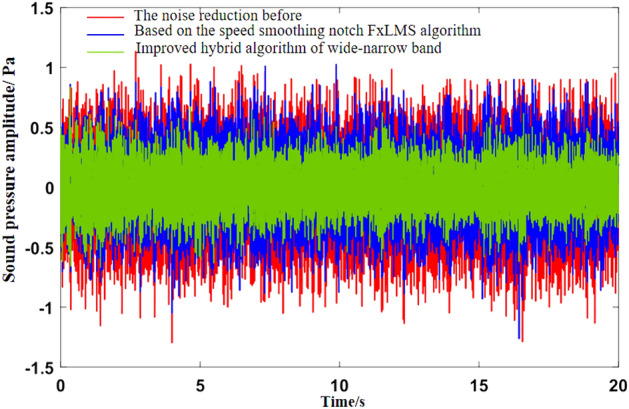
Noise reduction effect in time domain under uniform driving condition.

**Figure 13:**
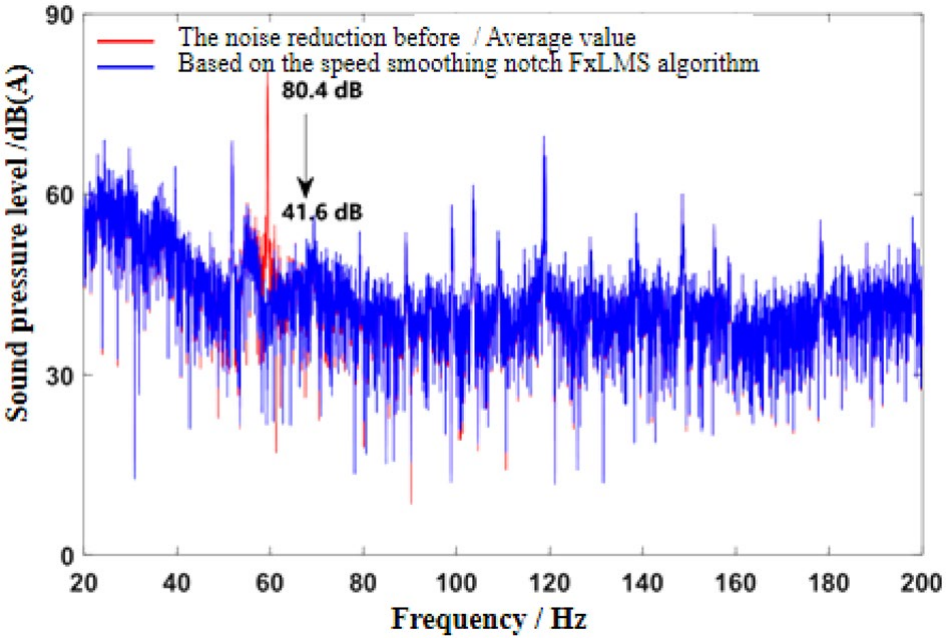
Effect of noise reduction in frequency domain under uniform speed condition (notch FxLMS algorithm based on speed smoothing).

**Figure 14:**
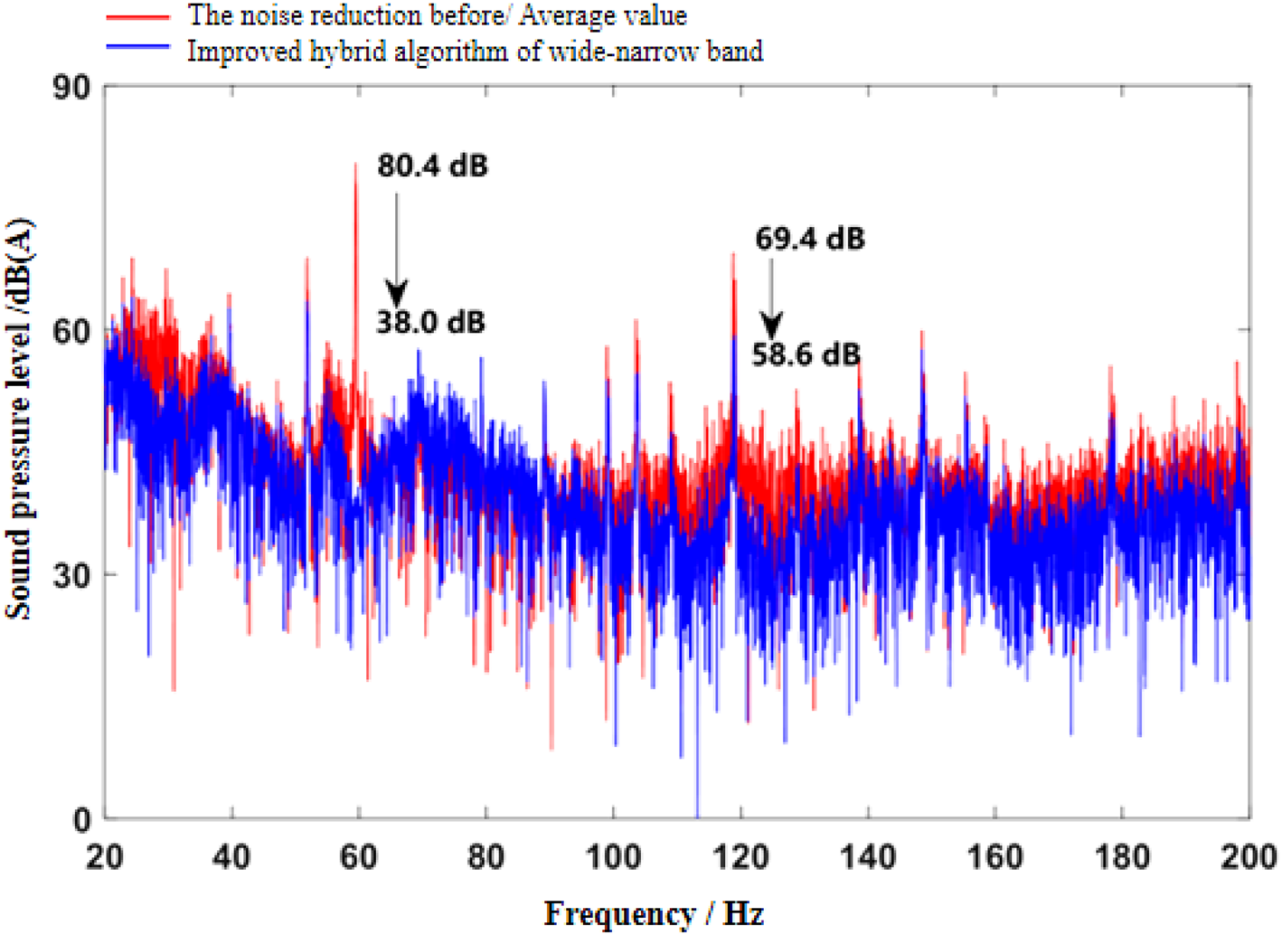
Noise reduction effect in frequency domain under uniform driving condition (improved wide-narrow-band hybrid ANC algorithm).

**Figure 15:**
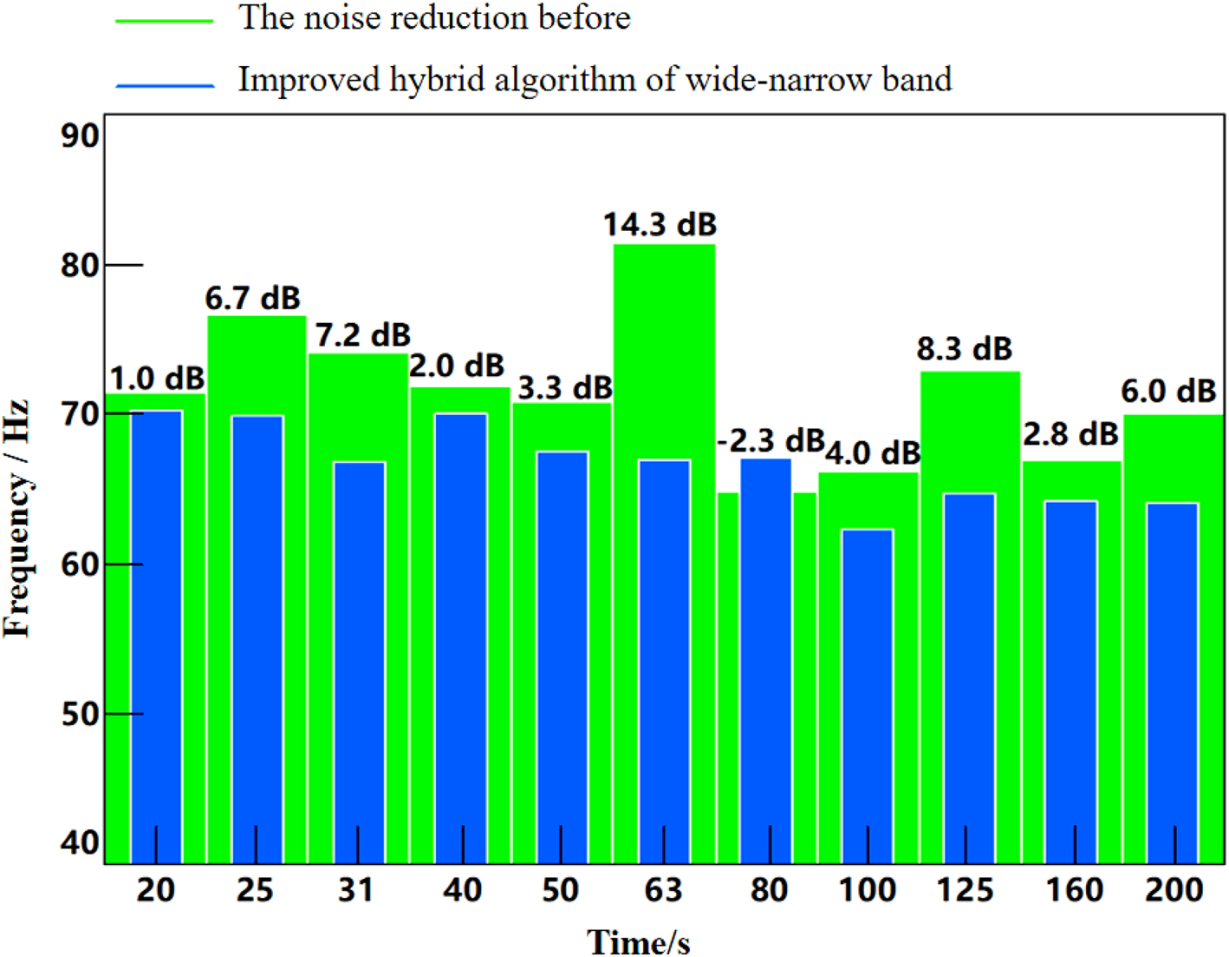
Effect diagram of 1/3 octave-frequency noise reduction under uniform driving speed (improved wide-narrow-band hybrid ANC algorithm).

In the MATLAB simulation model based on the notch FxLMS algorithm of speed smoothing and the improved wide-narrow-band hybrid ANC algorithm, the active noise control simulation of the collected noise signal is carried out. The selected algorithm parameters are shown in Table 4. The time domain noise reduction effect is shown in Fig. 12, and the frequency domain noise reduction effect is shown in Figs. 13, 14 and 15.

**Table 4:** Parameter values of the algorithm under 50 km/h uniform driving condition.

Algorithm	Parameters
Notch FxLMS algorithm based on speed smoothing	$\mu = 0.001,\lambda = 0.9999$
Improved wide and narrow band hybrid algorithm	$\mu_{S} = 0.001,\mu_{N} = 0.0001,\mu_{B} = 0.001,\phi = 0.001,b = 0.03,L_{0} = 3,L = 192$

As can be seen from Fig. 12, the convergence speed of FxLMS algorithm based on speed smoothing is faster than that of the improved broadband and narrowband hybrid ANC algorithm. The improved hybrid ANC algorithm has a higher steady noise reduction than the FxLMS algorithm based on speed smoothing. As can be seen from Fig. 13, notch FxLMS algorithm based on speed smoothing achieves a large noise reduction at the second-order frequency of the engine (59.5 Hz), and the sound pressure level drops from 80.4 to 41.6 dB, a decrease of 38.8 dB. It can be seen from Fig. 14 that the improved wide-narrow-band hybrid ANC algorithm has a better control effect on second-order noise of engine at 59.5 Hz. Moreover, a better noise reduction effect is achieved at the fourth order frequency of the engine (119 Hz). The sound pressure level at 59.5 Hz decreased from 80.4 to 38.0 dB, decreasing by 42.4 dB. The sound pressure level at 119 Hz decreased from 69.4 to 58.6 dB, decreasing by 10.8 dB. Figure 15 shows the noise reduction effect of 1/3 octave-frequency of the improved broadband and narrowband hybrid algorithm. As can be seen from the figure, sound pressure level at 80 Hz frequency increased by about 2.3 dB, and other frequency ranges achieved good noise reduction effects. The noise reduction of 14.3 dB and 8.3 dB is achieved for the engine order frequency, and the noise reduction of 1–8 dB is achieved for the other frequency range.

Figure 13 and 14 Two ANC active control algorithms, the secondary channel path has some shortcomings in noise reduction matching, mainly for the broadband signal noise reduction processing is weak. The effect of narrowband noise reduction is better, mainly due to the large cab space of commercial vehicles and the large microphone layout distance, which affects the noise reduction effect, and the other reason is the real-time and delay of the signal, which also has a certain impact on the broadband noise reduction effect.

### Acceleration condition verification

**Figure 16:**
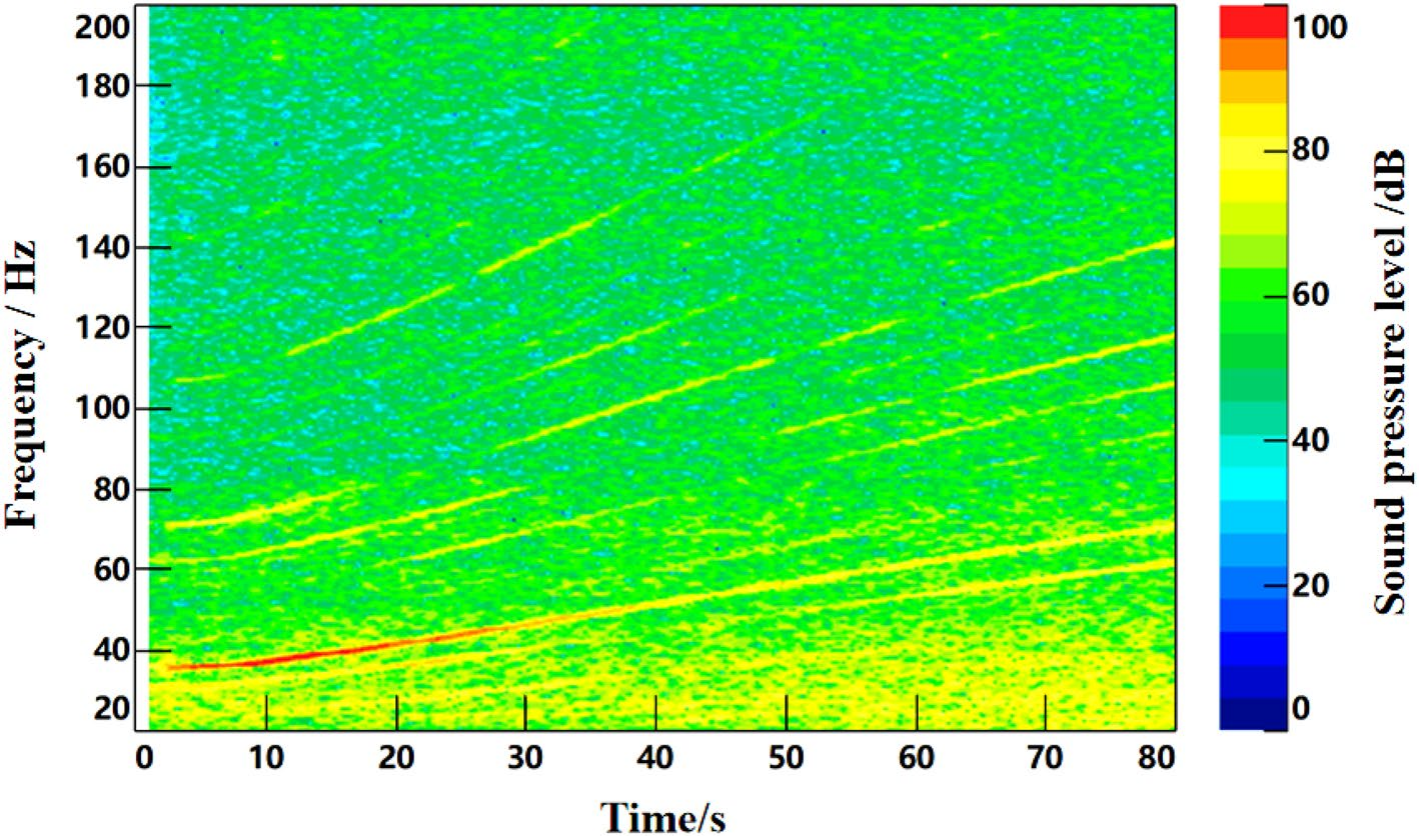
Time–frequency curve of interior noise under acceleration condition.

**Figure 17:**
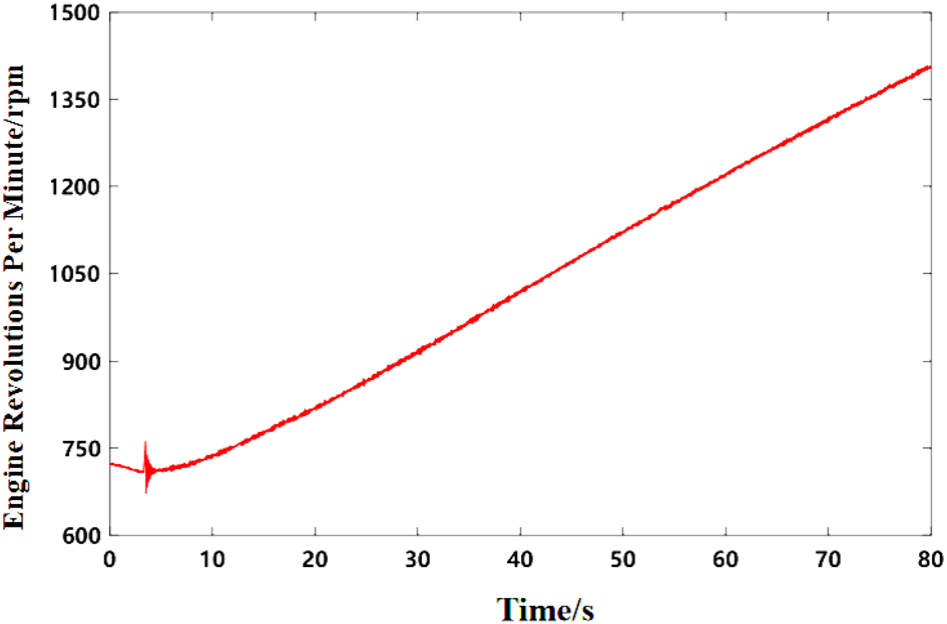
Engine speed curve under acceleration condition.

Under the same conditions, the noise and speed data of acceleration from 45 to 90 km/h were collected. The time–frequency diagram of noise is shown in Fig. 17, and the engine speed signal collected is shown in Fig. 17. As can be seen from Fig. 16, the second-order frequency value of the engine gradually increases as the engine speed continues to rise during acceleration. Figure 17 shows that the engine speed of the test vehicle increases from 740 to 1400 r/min under accelerated driving conditions.

**Figure 18:**
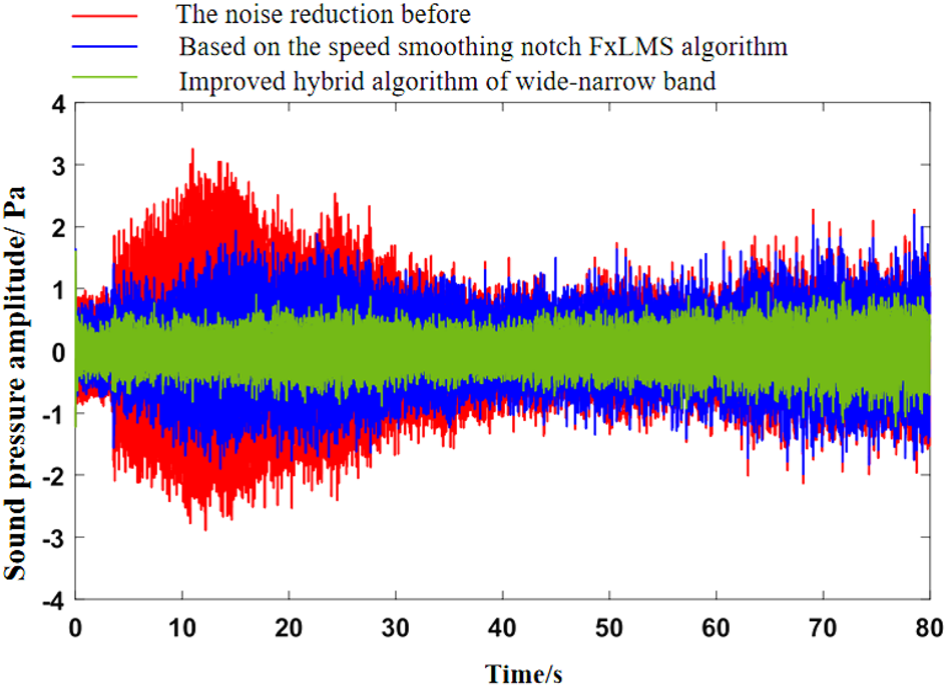
Time-domain noise reduction effect diagram of acceleration condition.

**Figure 19:**
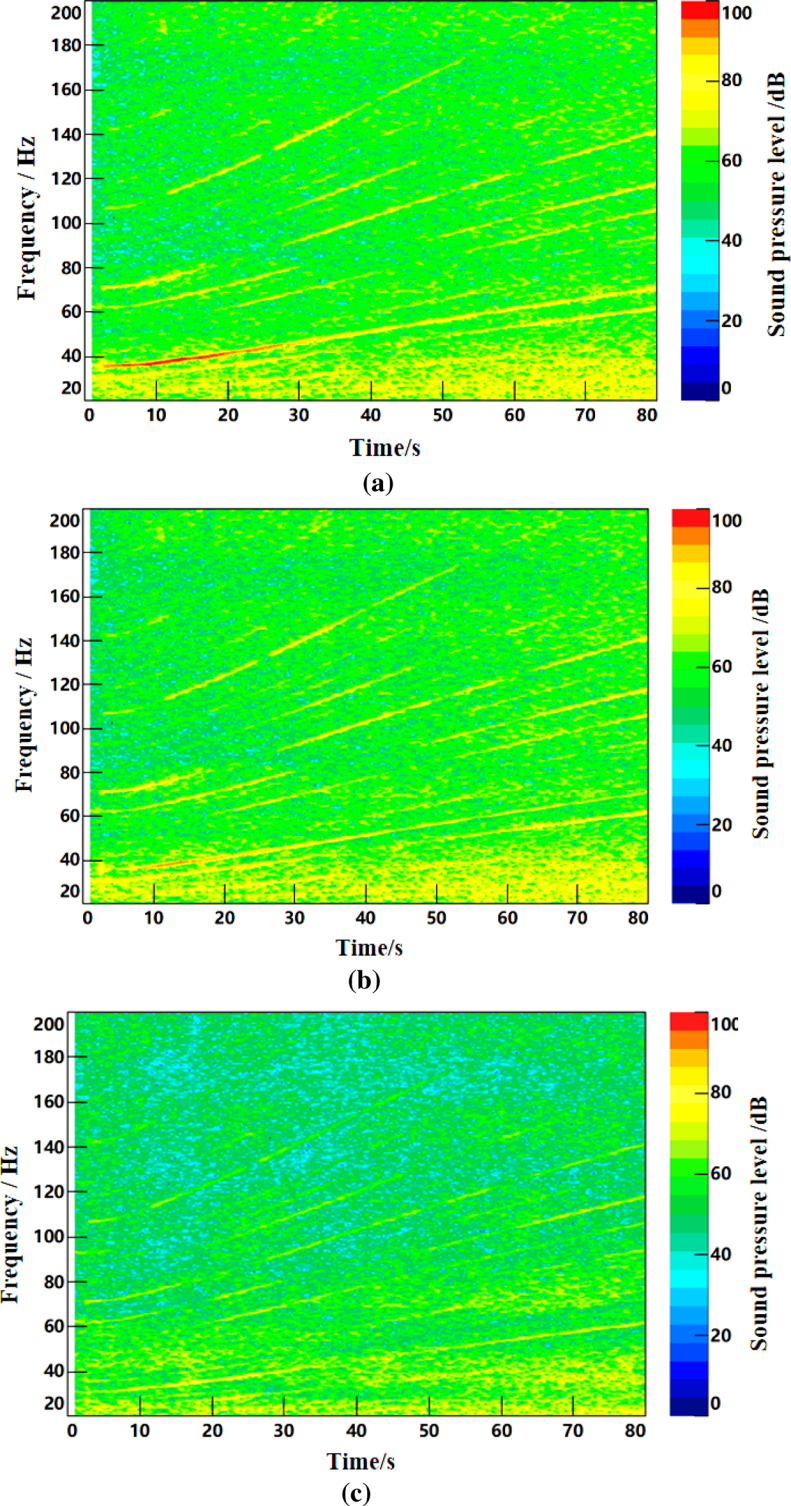
Time–frequency noise reduction effect diagram under acceleration condition. (**a**) Time–frequency diagram of interior noise before noise reduction. (**b**) Interior noise based on notch FxLMS algorithm for speed smoothing. (**c**) Improved wide and narrow band hybrid ANC algorithm for interior noise.

In the MATLAB simulation model based on the notch FxLMS algorithm of speed smoothing and the improved wide-narrowband hybrid ANC algorithm, active noise control simulation is carried out for the noise signals collected above. The selected algorithm parameters are shown in Table 5, time-domain noise reduction effect is shown in Fig. 18, time–frequency noise reduction effect is shown in Fig. 19.

**Table 5:** Algorithm parameter values in acceleration conditions.

Algorithm	Parameters
Notch FxLMS algorithm based on speed smoothing	$\mu = 0.001,\lambda = 0.9999$
Improved wide and narrow band hybrid algorithm	$\mu_{S} = 0.001,\mu_{N} = 0.0001,\mu_{B} = 0.001,\phi = 0.001,b = 0.03,L_{0} = 3,L = 192$

As can be seen from Fig. 18, notch FxLMS algorithm based on speed smoothing achieves good time-domain noise reduction in the first half of the acceleration process. However, the noise reduction effect of the second half is relatively poor. This is mainly because the latter half of the vehicle speed is higher, road noise, wind noise and other broadband components accounted for a larger proportion, so the overall noise reduction effect becomes worse. Comparatively, the improved hybrid ANC algorithm achieves high time-domain noise reduction in the whole acceleration process. This benefits from the control of broadband noise. It can be seen from Fig. 19 that, compared with the interior noise before noise reduction, notch FxLMS algorithm based on speed smoothing can produce a larger noise reduction for the second-order frequency noise of the engine during the whole acceleration process. However, there is no noise reduction effect for other orders of noise. Compared with FxLMS algorithm based on speed smoothing, the improved broadband and narrowband hybrid ANC algorithm has better control effect on engine second-order noise. At the same time, the noise of other order of engine (20–200 Hz) can be effectively controlled.

## Conclusion

Aiming at the problem of active noise control in commercial vehicle cab, an improved wide-narrow-band hybrid ANC algorithm was proposed. At the same time, the control effect of the proposed algorithm is analyzed and verified. The main research contents and conclusions are as follows:

1. On the basis of the existing active noise control algorithm, the notch FxLMS algorithm based on speed smoothing is proposed. By smoothing the speed signal, the adverse effect of engine speed fluctuation on the active noise control system is avoided. The method is combined with notch filter and FxLMS algorithm is used to update the weight coefficient of filter.
2. On the basis of FxLMS algorithm, an improved hybrid ANC algorithm with wide and narrow band is proposed. The algorithm consists of narrowband subsystem, broadband subsystem and signal separation subsystem. Narrow band subsystem adopts notch FxLMS algorithm with smooth speed. In broadband subsystem, FxLMS/F algorithm based on signal weighting is adopted to increase the weight of reference signal sampling, so as to enhance the control ability of active noise control system to broadband noise.
3. MATLAB models of FxLMS algorithm based on speed smoothing and ANC algorithm based on improved width and narrow band are established. The simulation results show that the FxLMS algorithm based on speed smoothing can effectively control the second-order engine noise in the cab of commercial vehicle. At idle speed, the sound pressure level of the FxLMS algorithm based on speed smoothing at the second order frequency (30 Hz) of the engine decreases from 89 dB before noise reduction to 52 dB, which decreases by 37 dB. Under uniform driving condition, the SPL at the second order frequency (59.5 Hz) of the engine decreases from 80.4 dB before noise reduction to 41.6 dB, decreasing by 38.8 dB. Under the acceleration condition, the SPL at the second order frequency of the engine decreases obviously during the whole acceleration process.
4. The limitation of the algorithm is determined by the space distance, which poses a challenge to the noise reduction effect of commercial vehicle cab in large space. The algorithm’s solution to the space distance problem is the direction of further development.

## Data Availability

The data of this study are available from the corresponding author upon request.
